# A Four‐Week Treatment With Dapagliflozin Is Associated With a Reduction in Insulin‐Stimulated Renal Cortical Glucose Uptake: A Post Hoc Analysis From a Pilot Study

**DOI:** 10.1111/dom.71026

**Published:** 2026-06-23

**Authors:** Shawn Gugliandolo, Cassandra Morciano, Andrea Guarneri, Gian Pio Sorice, Marta Giaccari, Luigi Cappannoli, Umberto Capece, Amelia Splendore, Adriana Avolio, Lorenzo Diego Greco, Patricia Iozzo, Rossana Moroni, Alfredo Pontecorvi, Maria Lucia Calcagni, Francesco Burzotta, Domenico D'Amario, Filippo Crea, Lucia Leccisotti, Francesca Cinti

**Affiliations:** ^1^ Centro Malattie Endocrine e Metaboliche, Dipartimento di Scienze Mediche e Chirurgiche Fondazione Policlinico Universitario A. Gemelli IRCCS and Università Cattolica del Sacro Cuore Rome Italy; ^2^ Unità di Medicina Nucleare Fondazione Policlinico Universitario A. Gemelli IRCCS Rome Italy; ^3^ Sezione di Medicina Interna, Endocrinologia, Andrologia e Malattie Metaboliche, Dipartimento di Medicina di Precisione e Rigenerativa e Area Jonica (DiMePRe‐J) Università Degli Studi di Bari “Aldo Moro” Bari Italy; ^4^ UOC Nephrology and Dialysis, ASL Roma 1, and Division of Nephrology and Dialysis Bambino Gesù Children’s Hospital, IRCCS Rome Italy; ^5^ UOC di Cardiologia, Dipartimento di Scienze Cardiovascolari Fondazione Policlinico Universitario A. Gemelli IRCCS and Università Cattolica del Sacro Cuore Rome Italy; ^6^ Istituto di Fisiologia Clinica, Consiglio Nazionale Delle Ricerche (CNR) Pisa Italy; ^7^ Biostatistician—Independent Researcher (PhD) Rome Italy; ^8^ Dipartimento di Medicina Traslazionale Università del Piemonte Orientale Novara Italy; ^9^ Sezione di Medicina Nucleare, Dipartimento di Scienze Radiologiche Ed Ematologia Università Cattolica del Sacro Cuore Rome Italy

## Abstract

**Introduction and Objective:**

Treatment with sodium‐glucose cotransporter‐2 inhibitors (SGLT‐2i) reduces the risk of both cardiovascular and renal outcomes in type 2 diabetes (T2D). The specific mechanism by which this happens has not yet been clarified. Recent findings indicate that, in obese individuals, the kidney exhibits lower 18‐fluorodeoxyglucose ([18F]‐FDG) uptake rates compared to lean individuals, possibly due to insulin resistance. This post hoc analysis from our pilot study DAPAHeart aims to evaluate changes in cortical renal [18F]‐FDG uptake in T2D patients following short‐term treatment with dapagliflozin.

**Methods:**

In our DAPAHeart trial, a single‐centre, 4‐week, prospective, double‐blind, controlled study, we enrolled T2D patients with stable coronary artery disease and preserved glomerular filtration rates. Participants were randomly assigned in a 1:1 ratio to receive either dapagliflozin (10 mg daily) or a placebo. PET scans using [18F]‐FDG were performed during a hyperinsulinemic‐euglycemic clamp (HEC) to measure regional FDG uptake before and after the 4‐week treatment with SGLT‐2i.

**Results:**

After 4 weeks of treatment, the between‐group comparison (Mann–Whitney *U* test on change‐from‐baseline values) did not reach statistical significance for any SUV parameter. Nevertheless, a consistent directional pattern in renal standardised uptake value (SUV) was noted across all parameters, with reductions observed in the dapagliflozin group and stable or slightly increased values in the placebo group. The within‐group analysis revealed a significant reduction in SUV_peak in the dapagliflozin group (*p* = 0.028), while no significant change was detected in the placebo group.

**Conclusion:**

Although the primary between‐group comparison did not reach statistical significance, likely due to the limited sample size (*n* = 7 per group), a consistent directional pattern was observed across all renal SUV parameters, with reductions in the dapagliflozin group and stable or slightly increased values in the placebo group. These findings suggest decreased glucose uptake in an insulin‐stimulated state (HEC) in the dapagliflozin group. Although the causes of this reduction require further investigation, these preliminary findings support the hypothesis that SGLT‐2 inhibition may reduce tubular energy requirements and glucose uptake, potentially contributing to the preservation of kidney function.

**Abbreviations**
[18F]‐FDG: 2‐deoxy‐2‐[18F]fluoro‐D‐glucose
CT: computed tomography
GLUT: glucose transporters
HEC: hyperinsulinemic‐euglycemic clamp
PET: positron emission tomography
SGLT: sodium‐glucose cotransporter
SGLT‐2i: sodium‐glucose cotransporter‐2 inhibitors
T2D: type 2 diabetes

## 1 Introduction

Individuals with diabetes are at elevated risk of developing chronic kidney disease (CKD), which in turn increases the likelihood of kidney failure, cardiovascular disease and premature death, intensifying diabetes burden. Insulin resistance is a key determinant in the worsening of kidney function, mostly due to PI3K/Akt signalling defects and mitochondrial dysfunction [1].

Rebelos and colleagues have already demonstrated that the renal cortex acts as an insulin‐sensitive tissue [2]. In their study, they report that both the medullary and cortical regions show higher glucose uptake in lean subjects compared to obese subjects, but only the renal cortex shows increased glucose uptake in response to insulin stimulation. Therefore, insulin resistance specifically affects the renal cortex, leading to significantly reduced glucose uptake rates [2]. This finding was confirmed by a recent study [3], which showed that modest reductions in sedentary behaviour increased renal cortical glucose uptake and were positively associated with insulin sensitivity while being negatively associated with body mass index (BMI) and circulating free fatty acid levels.

Large cardiovascular and renal outcome trials have established the role of sodium‐glucose cotransporter‐2 inhibitors (SGLT‐2i) in reducing major cardiovascular events (MACEs), hospitalisation for heart failure, all‐cause mortality and progression to end‐stage kidney disease. SGLT2‐i act at the level of proximal convoluted tubule (PCT) and have been shown to slow decline in kidney function in patients with CKD (DAPA‐CKD [4], EMPA‐KIDNEY [5] and CREDENCE [6]) as well as in those without CKD (EMPA‐REG OUTCOME [7], DECLARE‐TIMI 58 [8], CANVAS [9]). Collectively, these findings have expanded the indications for SGLT‐2i beyond glucose‐lowering, highlighting their role in providing cardiorenal protection [10]. Moreover, increasing evidence points to the impact of these new therapeutic options on different organs [11], underscoring the need for a deeper understanding of regional glucose metabolism.

In the DAPAHeart Trial, a single‐centre, 4‐week, prospective, double‐blind, randomised controlled study designed in 2016, we enrolled type 2 diabetes subjects with chronic coronary syndrome, without heart failure and with preserved renal function [12]. Patients underwent PET scans using 2‐deoxy‐2‐[18F]fluoro‐D‐glucose ([18F]‐FDG) during a hyperinsulinemic‐euglycemic clamp (HEC) to measure regional FDG uptake before and after the 4‐week treatment with the SGLT‐2i or placebo. The study aimed to investigate whether improvements in myocardial metabolism mediate the cardiovascular protective effects of dapagliflozin. We demonstrated that dapagliflozin increases coronary flow reserve (CFR) by 35%, an effect potentially driven by the observed 19% reduction in epicardial adipose tissue (EAT) thickness [13].

In the light of recent evidence that the renal cortex is an insulin‐sensitive tissue and that renal glucose uptake is globally reduced in obese subjects compared to lean individuals [2], we decided to conduct a sub‐analysis of the DAPAHEART trial focusing on renal cortical glucose uptake, so as to characterise the pathophysiological defects associated with diabetes and to explore additional mechanisms underlying the renal benefits induced by SGLT‐2i.

To this end, the primary objective of our post hoc sub‐analysis is to assess any changes in standardised uptake value peak activity concentration (SUV peak) following 4 weeks of treatment (or placebo) in the two patient groups under investigation.

## 2 Research Design and Methods

Further details as to the study design and detailed imaging can be found in our previously published protocol paper [14]. The study was conducted in our teaching hospital Fondazione Policlinico Universitario Agostino Gemelli IRCCS, approved by the local ethics committee (study protocol code GIA‐DAP‐16‐005) and registered at eudract.ema.europa.eu (EudraCT No. 2016 003614‐27) and ClinicalTrials.gov (NCT 03313752). Informed, written consent was obtained from all participants. In this phase III, single‐centre, randomised, two‐arm, parallel‐group, double‐blind, placebo‐controlled study, we enrolled 16 participants with type 2 diabetes and chronic coronary syndrome (coronary stenosis ≥ 30% and < 80%; with or without previous percutaneous coronary intervention in the preceding 6 months), without heart failure and with preserved renal function (estimated glomerular filtration rates eGFR > 60 mL/min). The participants were randomly assigned on a one‐to‐one ratio to receive a 4‐week treatment with either dapagliflozin or a placebo, alongside their standard medication.

Regional glucose uptake was assessed using a whole body FDG‐PET/CT during a hyperinsulinemic‐euglycemic clamp, both at baseline and after 4 weeks.

### 2.1 PET Imaging and Analysis

PET/CT was performed at baseline and after 4 weeks of treatment using a 3D PET/CT scanner (Biograph mCT, Siemens Healthcare).

### 2.2 FDG PET/CT During Hyperinsulinemic‐Euglycemic Clamp

Additional details on imaging and metabolic procedures can be found in the protocol paper [14]. Briefly, all PET/CT studies were conducted after an overnight fast, and before undergoing the PET/CT scan, patients were instructed to empty their bladder.

We employed gold standard techniques to assess insulin sensitivity, including the hyperinsulinemic‐euglycemic clamp, which involves the simultaneous administration of intravenous insulin and glucose to achieve an euglycemic state. After at least 60 min of hyperinsulinemic euglycemia, a 50‐min dynamic PET/CT scan was performed after the intravenous administration of FDG (185 MBq). A CT transmission scan was acquired from the vertex to the mid‐thigh for photon attenuation correction and anatomical localisation. Regions of interest (ROIs) were manually drawn on the fused PET/CT images using all three transaxial, sagittal, and coronal planes to identify renal cortical glucose uptake. Standard uptake values (SUVs) were measured and expressed as mean (SUVmean), maximum (SUVmaximum) and peak (SUVmean) activity concentration. Measurements were taken by two independent nuclear medicine physicians unaware of the results, yielding an interobserver variability of less than 5%.

## 3 Statistical Analysis

The sample has been described in its clinical and demographic characteristics applying descriptive statistics techniques. Qualitative variables have been described with absolute frequencies and percentage; quantitative variables have been summarised with median and Interquartile Range (IQR). Given the limited sample size (*n* = 7 per group), non‐parametric tests were employed for all statistical analyses. Particularly, baseline comparisons have been conducted applying the Mann Whitney *U* test. The primary outcome measure, SUV_peak, was evaluated using a change‐from‐baseline approach, whereby an individual difference score (ΔSUV_peak = SUV_peak post‐treatment − SUV_peak baseline) was computed for each patient. Between‐group differences in ΔSUV_peak were then assessed using the Mann–Whitney *U* test. The same approach was applied to the secondary parameters SUV_max and SUV_mean.

This analytical approach was preferred over a mixed‐design repeated‐measures ANOVA for two reasons. First, it directly captures the treatment effect of interest, namely, whether the magnitude of change over time differs between the DAPA and placebo groups and is therefore mathematically equivalent to testing the group × time interaction term in a two‐way mixed ANOVA. Second, it avoids the parametric assumptions of the ANOVA framework (normality, sphericity, homogeneity of variance), which cannot be reliably assessed in small samples and are particularly prone to violation when group sizes are limited.

Within‐group changes from baseline to 4 weeks were assessed using the Wilcoxon signed‐rank test, applied separately to each group for descriptive purposes. It should be noted that these analyses do not constitute the primary statistical test and must be interpreted with caution, given the very limited statistical power associated with a sample size of *n* = 7. A two‐tailed *p* value of less than 0.05 was considered statistically significant for all tests. All statistical analyses were performed using R (4.4.1).

## 4 Results

From the original DAPAHeart trial enrolling 16 patients, a total of 14 patients were included in the present sub‐analysis (dapagliflozin *n* = 7, specifically seven males; placebo *n* = 7, specifically three females and four males) due to the following reasons: one patient has been excluded due to extravasation of the radiopharmaceutical, which compromised the reliability of the kinetic quantification, while for the other patient we had a suboptimal abdominal scan quality.

The two groups presented no statistically significant differences at baseline (Table 1) with respect to a set of clinical and demographic variables.

**TABLE 1:** Baseline clinical and demographic characteristics of patients included in the sub‐analysis.

	PLACEBO, *N* = 7		DAPA, *N* = 7		Baseline comparison *p*
	Median	IQR	Median	IQR	
Age	69.0	63–72	67.0	55–71	0.71
Diabetes duration	14.0	8–20	10.0	5–16	0.456
SUV_peak_pre	2.0	1.69–2.21	1.6	1.49–2.42	0.71
SUV_max_pre	2.2	1.93–2.36	1.9	1.69–3.23	0.902
SUV_mean_pre	1.4	1.18–1.63	1.2	1.05–1.9	0.805
M_PRE	3.1	2.33–3.22	2.1	1.71–4.27	0.383
HBA1c (%)	8.3	7.5–8.5	7.6	7.5–8.5	0.71
BMI (kg/m^2^)	28.7	26.5–29.4	27.3	25.3–31.4	0.71
WEIGHT (kg)	85.0	68–88	83.0	77–89	0.902
eGFR_PRE ml/min/1.73 m^2^	95.5	88.6–98.1	92.3	75.6–101	0.902

Medications	Placebo (*n* = 7) (%)	Dapagliflozin (*n* = 7) (%)
Metformin	7 (100)	6 (85)
DPP‐4i	4 (57)	2 (29)
GLP1‐RA	2 (29)	1 (14)
Basal insulin	2 (29)	2 (29)
Sulfonylurea	1 (14)	1 (14)

From a purely descriptive standpoint, after 4 weeks of treatment, SUV_peak decreased in both groups over the study period. Specifically, in the dapagliflozin group, six patients out of seven exhibited a reduction in SUV_peak between baseline and 4 weeks; one patient exhibited an increase in SUV_peak. In the placebo group, one patient showed no change between the baseline and 4‐week measurements, while two patients out of seven demonstrated an increase. When considering the mean percentage change, the dapagliflozin group showed a 15.2% reduction in SUV_peak, compared to a 4.8% reduction observed in the placebo group (Table 2).

**TABLE 2:** Descriptive statistics for SUV_peak, SUV_max and SUV_mean (change‐from‐baseline).

	PLACEBO, *N* = 7		DAPA, *N* = 7	
	Median	IQR	Median	IQR
SUV_peak_pre	2.0	1.69–2.21	1.6	1.49–2.42
SUV_max_pre	2.2	1.93–2.36	1.9	1.69–3.23
SUV_mean_pre	1.4	1.18–1.63	1.2	1.05–1.9
SUV_peak_post	1.8	1.7–1.9	1.5	1.3–1.9
SUV_max_post	2.0	1.97–2.3	1.8	1.6–2.5
SUV_mean_post	1.4	1.3–1.4	1.3	1.1–1.5

The primary between‐group comparison, based on the Mann–Whitney *U* test applied to the differences between baseline and 4 weeks SUV_peak values, did not demonstrate a statistically significant difference between the dapagliflozin and placebo groups (*U* = 21, *p* = 0.710). See Table 3 for details where for completeness, non‐significant *p* values associated with measurements not pertaining to the primary study objective are also reported.

**TABLE 3:** Between groups comparisons.

	PLACEBO, *N* = 7 (median; IQR)	DAPA, *N* = 7 (median; IQR)	Mann Whitney *U* test *p*, PLACEBO vs. DAPA
Delta_SUV_Max	0.04 (−0.4; 0.3)	−0.3 (−1.14; 0)	0.383
Delta_SUV_mean	0.05 (−0.2; 0.3)	−0.1 (−0.6; 0.14)	0.535
Delta_SUV_peak	−0.3 (−0.5; 0.2)	−0.2 8 (0.5; −0.2)	0.710

Although none of the comparisons reached statistical significance, a consistent directional pattern was observed across all three parameters, with reductions in the dapagliflozin group and stable or slightly increased values in the placebo group.

**FIGURE 1:**
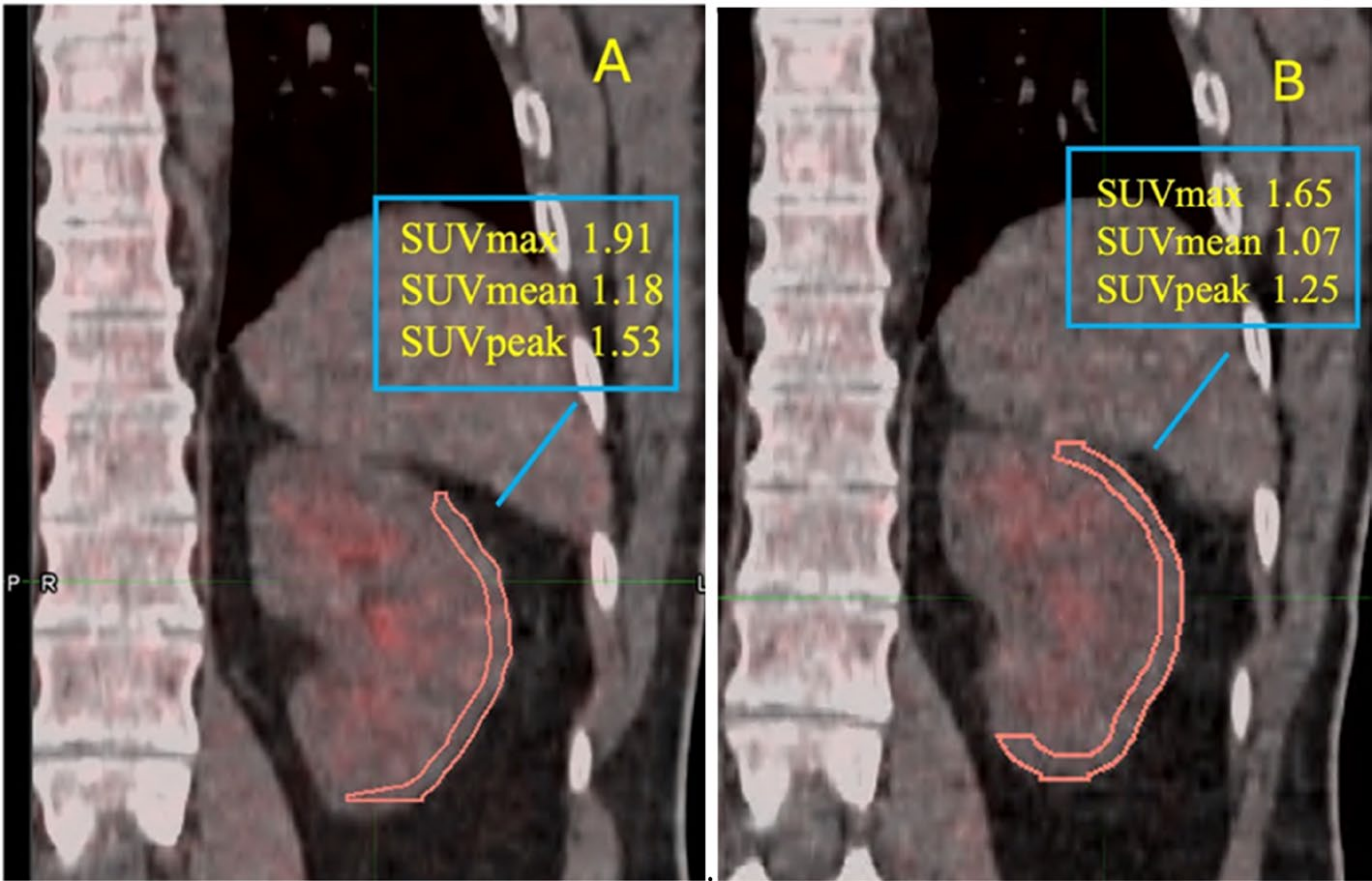
Visual representation of the volume of interest (VOI), approximately equal to 30 cm^3^ (cc), placed on the renal cortex, in order to avoid the interference of physiological glucose activity by urinary excretion. SUV is expressed as MegaBequerel/g (MBq/g). Representative image of a patient of DAPA group at baseline (A) and after 4 weeks (B).

**FIGURE 2:**
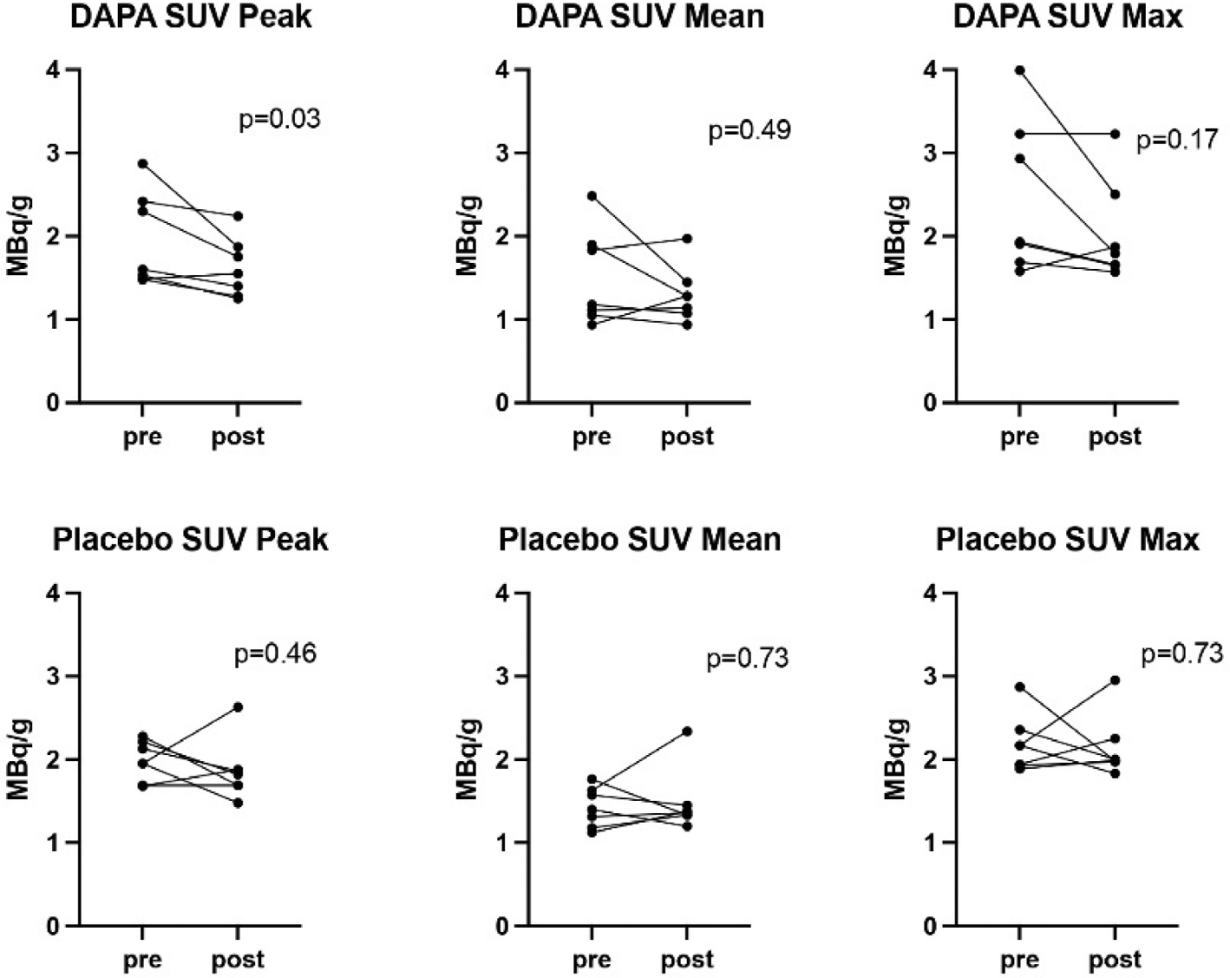
Kidney cortical glucose uptake: within‐group changes of insulin‐stimulated kidney cortical glucose uptake at baseline vs. 4 weeks of dapagliflozin treatment (DAPA) (first row) or placebo (second row). SUV is expressed as MegaBequerel/g (MBq/g).

Within‐group changes highlighted, in the dapagliflozin group, a statistically significant reduction in SUV_peak was observed between baseline and 4 weeks (*p* = 0.028) (Figures 1 and 2). No significant within‐group change was detected in the placebo group. See Table 4 for details where for completeness, non‐significant *p* values associated with measurements not pertaining to the primary study objective are also reported.

**TABLE 4:** Within groups comparisons.

	PLACEBO, *N* = 7	DAPA, *N* = 7
SUV_peak_pre vs. SUV_peak_post (MBq/g)	0.463	0.028
SUV_mean_pre vs. SUV_mean_post (MBq/g)	0.735	0.498
SUV_Max_pre vs. SUV_Max_post (MBq/g)	0.735	0.173

**FIGURE 3:**
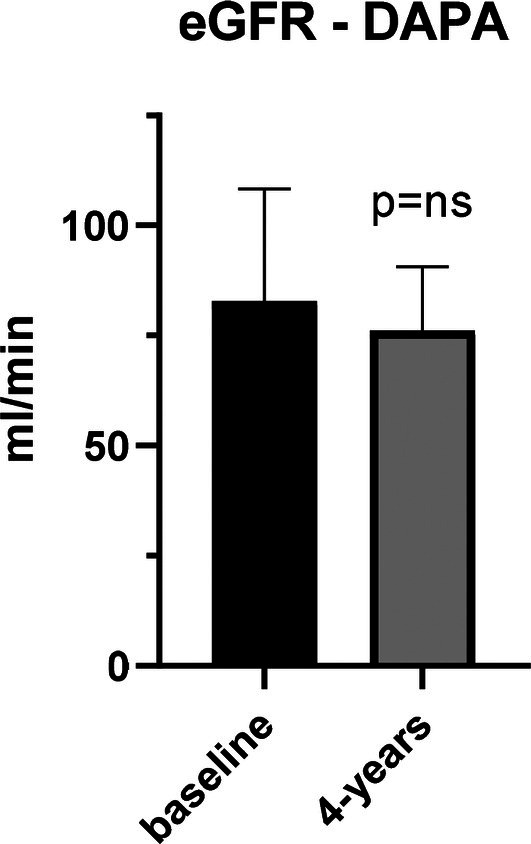
eGFR baseline and after 4 years of dapagliflozin treatment.

After completion of the 4‐week trial, patients in the placebo group also initiated treatment with dapagliflozin and both groups were therefore followed‐up for 4 years. An interesting finding was that the eGFR remained stable over the 4 years (pre‐therapy value was 82.9 ± 9.6 mL/min, and 76.2 ± 5.47 mL/min after 4 years (*p* = ns)) (Figure 3).

## 5 Discussion

Our data on the kidney show that insulin‐stimulated cortical FDG uptake, expressed as standardised uptake value peak activity (SUV Peak), showed a consistent directional reduction after a 4‐week treatment with dapagliflozin compared to placebo, across all SUV parameters, though the primary between‐group comparison did not reach statistical significance, most likely due to the limited sample size. Notably, a significant within‐group reduction in SUV_peak was observed in the dapagliflozin group, with no corresponding change in the placebo group, lending directional support to a treatment‐related effect on renal cortical glucose uptake.

Although the precise mechanisms by which SGLT‐2 inhibitors reduce renal cortical uptake are not yet fully elucidated, several hypotheses exist in the literature to account for this effect. We have to take into consideration that the reduction in insulin‐stimulated cortical FDG uptake, in terms of SUV peak, may be a direct effect of SGLT‐2i on FDG handling at a tubular level, leading to lower intratubular tracer concentration. We can argue that glucose reabsorbed via SGLT2 is predominantly translocated across the basolateral membrane into the circulation and, accordingly, it is not significantly phosphorylated for glycolytic metabolism, as its principal function is not to serve as an energy substrate at the tubular level. Therefore, the phenomenon may not simply reflect the inhibition of SGLT‐2 at a tubular level and within a nephroprotective context, we can hypothesise that decreased 18F‐FDG uptake (and indirectly glucose utilisation) is associated with a reduction of glucose energetic requirement ultimately relying on different substrates. The metabolic reprogramming proposed by Ferrannini and colleagues [15] suggests that by promoting glycosuria, and therefore decreasing plasma glucose levels, SGLT‐2 inhibition leads to a reversal in glucotoxicity [16]. The decreased insulin levels and increased glucagon concentration stimulate lipolysis and induce the release of free fatty acids (FFAs). In the liver, FFAs undergo β‐oxidation, producing acetyl‐CoA, which is used to support ketogenesis, providing a fuel‐efficient substrate for the heart and the kidney [17]. Ketones may serve as efficient energy substrates by enhancing mitochondrial ATP production and thus reducing fatty acid oxidation, an oxygen‐intensive process that can generate harmful reactive oxygen species [18]. Concurrently, SGLT2‐i induce glycosuria reducing hyperfiltration and tubular activity, the main determinants of renal energy and oxygen consumption [19]. Each molecule of glucose reabsorbed by the kidney indirectly costs energy because of the Na^+^/K^+^ ATPase pumps on the tubules. The lower gradient between the inner and outer compartments, leads to reduced ATP consumption [20], which in the long term can contribute to renal protection. The kidneys and the heart are among the most energetically active organs, due to their high metabolic demands [21]. Within the kidney, PCTs in particular, are the leading segment of the nephron responsible for both producing and consuming energy, given their pivotal role in reabsorption, secretion and endocrine homeostasis. Under normal circumstances, and akin to cardiomyocytes, these cells exhibit a marked inability to perform glycolysis and depend entirely on fatty acid oxidation (FAO), due to their location in the renal cortex, which has a high oxygenation level. Nonetheless, PCTs are capable of switching to glycolysis (and therefore anaerobic metabolism) in pathological conditions such as ischemia [22]. Persistent loss of FAO has been observed in renal biopsies of patients with CKD and is associated with kidney fibrosis. This finding indicates that metabolic reprogramming represents a crucial moment in CKD development, as the reliance on glycolysis triggers pro‐fibrotic pathways in tubular cells [23]. We speculate that SGLT‐2i limit the availability of intracellular glucose within PCTs, thereby compelling them to engage in FA oxidation thus preventing renal fibrosis. In addition, perirenal adipose tissue may play a role in renal function [24] and the DAPAHEART trial has generated a working hypothesis on the role of different adipose tissue depots. In fact, we observed a significant reduction in epicardial adipose tissue thickness and glucose uptake, which we interpreted as reflecting a decrease in the inflammatory state of this depot in diabetes. This reduction was associated with an improvement in coronary flow reserve due to the relief of microvascular dysfunction. We therefore evaluated perirenal adipose tissue to investigate possible associations underlying the reduction in cortical renal uptake. In fact, perirenal visceral adiposopathy is a key contributor to insulin resistance, a predictor of renal dysfunction in type 2 diabetes and a mediator of deleterious atherosclerotic effects [25, 26]. In our previous work, we found that perirenal thickness, expressed in centimetres, remained similar between the two groups although there was a non‐significant trend towards a higher perirenal glucose uptake rate in the dapagliflozin group [13]. We can hypothesise that, in the early stage of treatment, the increase in perirenal glucose uptake, suggesting improved metabolic activity, precedes the change in its thickness. Indeed, the observed changes in epicardial adipose tissue suggest that depot‐specific metabolic responses are already actively engaged within the first weeks of therapy. Not all fat depots respond at the same rate due to differences in cellular plasticity and relative proportions of metabolically active brown/beige adipose tissue [27]. This localised metabolic improvement in a crucial visceral fat depot may therefore drive the initial functional benefits, such as the observed increase in coronary flow reserve, preceding more widespread systemic changes [28]. Indeed, we are planning a follow‐up assessment of the perirenal adipose tissue after 4 years of treatment to confirm the hypothesised differences in metabolic timing across various depots.

In this regard, during our long term follow‐up spanning 4 years, both the groups were on dapagliflozin therapy and the eGFR remained stable over the 4 years. It is known that the normal age‐related decline in eGFR is around 1 mL/min per year when eGFR is above 60 mL/min and may reach 3–5 mL/min in type 2 diabetes patients [29]. Our data show that treatment with dapagliflozin is associated with stable eGFR values, with an eGFR slope comparable to that observed in the general population.

### 5.1 Limitations and Strengths of the Study

Limitations of the present study must be acknowledged. Most notably, the sample size was small (*n* = 7 per group), primarily due to recruitment constraints imposed by COVID‐19 restrictions and the complexity of the screening procedures. This limited statistical power (a post hoc power analysis with the Mann Whitney *U* test—one tailed, alpha = 0.05—revealed a statistical power of approximately 17%, under the assumption of a medium effect size with Cohen's *d* = 0.5) likely accounts for the absence of significance in the primary between‐group analysis, as the consistent directional pattern observed across all SUV parameters together with the significant within‐group reduction in SUV_peak in the dapagliflozin group (*p* = 0.028) suggests that a larger, adequately powered cohort would have been more likely to detect a statistically significant treatment effect. The study was designed in 2016, before robust evidence on renal protection had come to light; consequently, renal outcomes were not included among the primary endpoints. Data collection was confined to the cortical region of the kidney as detailed assessment of the medulla was not feasible, requiring correction of fractional uptake rates for residual FDG in the urine. [18F]FDG‐PET can be a reliable tool to assess renal glucose metabolism in humans. Over the years this technique has been neglected due to the complex structure of the organ. Following [18F]FDG administration, the tracer is filtered by the glomerulus but is not fully reabsorbed by SGLT‐1 and SGLT‐2 due to its low affinity [30], leading to partial accumulation in the urine.

On the other hand, our study also has many strengths. Gold standard techniques were used to assess whole body insulin sensitivity (HEC) and regional glucose metabolism in terms of uptake (18F‐FDG‐PET/CT) [31]. Notably, we were able to observe a significant decrease in cortical renal uptake after only 4 weeks of treatment.

## 6 Conclusions

A 4‐week treatment with dapagliflozin is associated with a consistent directional reduction in insulin‐stimulated renal cortical FDG uptake, expressed as SUVpeak, across all SUV parameters. Although the primary between‐group comparison did not reach statistical significance (Delta_SUV_peak: *p* = 0.710), likely due to the limited sample size, the directional pattern observed consistently across all SUV measures suggests a biologically plausible treatment effect. It is notable that these directional changes emerge after a relatively short treatment period of only 4 weeks. Although the mechanisms underlying this reduction require further investigation, SGLT‐2 inhibition is thought to reduce glucotoxicity, and promote a metabolic shift towards FFA oxidation and ketogenesis. This shift is accompanied by a reduction in ATP consumption driven by decreased Na^+^/K^+^‐ATPase pump activity and possibly by a lower pro‐inflammatory milieu due to a decrease in perirenal adipose tissue thickness (an effect not yet observed at the 4‐week follow‐up). These processes may lower tubular energy requirements and contribute to the preservation of kidney function, as confirmed by eGFR data over the four‐year follow‐up.

## Author Contributions

Francesca Cinti, Gian Pio Sorice, Lucia Leccisotti and Patricia Iozzo designed the trial. Andrea Guarneri, Lucia Leccisotti and Maria Lucia Calcagni described and reviewed PET/CT procedures. Luigi Cappannoli performed, and Domenico D'Amario, Francesco Burzotta and Filippo Crea reviewed the cardiological procedures. Francesca Cinti, Shawn Gugliandolo, Cassandra Morciano, Umberto Capece, Amelia Splendore, Adriana Avolio, Alfredo Pontecorvi and Lorenzo Diego Greco performed and followed the metabolic procedures. Lucia Leccisotti and Andrea Guarneri performed the PET/CT scans. Shawn Gugliandolo, Cassandra Morciano, Andrea Guarneri, Gian Pio Sorice, Marta Giaccari, Lucia Leccisotti and Francesca Cinti were the major contributors in analysing data and writing the manuscript. Rossana Moroni performed the statistical analysis. All authors have read and approved the final manuscript.

## Funding

C.M. was supported by the PRIN PNNR 2022, Prot. P2022445ES, CUP: J53D23017390001 from the MUR—Ministry of University and Research.

## Conflicts of Interest

The authors declare no conflicts of interest.

## Data Availability

The data availability statement will be provided as per the request.
